# Membranous Nephropathy Secondary to Syphilis

**DOI:** 10.1155/crin/9592129

**Published:** 2026-09-23

**Authors:** Ifeoluwa Stowe, Shreya R. Saxena, Riya Paluri, Damodar R. Kumbala

**Affiliations:** ^1^ Division of Nephrology, Beth Israel Deaconess Medical Center and Harvard Medical School, Boston, Massachusetts, USA, harvard.edu; ^2^ Goyal Eye Institute, New Delhi, India; ^3^ Boston University School of Medicine, Boston, USA, bu.edu; ^4^ Renal Associates of Baton Rouge, Baton Rouge, Louisiana, USA

**Keywords:** membranous nephropathy, nephrotic syndrome, renal disease, syphilis

## Abstract

Membranous nephropathy (MN) is one of the most common causes of nephrotic syndrome in the adult population. Although the majority of the cases of MN are without any inciting event, about a third are caused or associated with immune diseases, infectious diseases, and drugs and toxins. There have been very few cases of association of syphilis and MN. We describe this rare infectious cause of MN in a 41‐year‐old patient who presented with nephrotic syndrome. The patient was diagnosed with secondary syphilis. He was treated successfully with complete resolution of his nephrotic picture. A thorough clinical history and physical examination which resulted in a suspicion for sexually transmitted disease led us to the successful diagnosis and treatment of this rare condition.

## 1. Introduction

Membranous nephropathy (MN) is one of the most common causes of adult nephrotic syndrome. The vast majority of cases of MN are idiopathic, with secondary forms accounting for approximately 20% [1]. Syphilis is a rare infectious cause of MN. Nephrotic syndrome, nonnephrotic range proteinuria, microscopic hematuria, hypertension, and renal failure are different forms of clinical presentation of MN [1]. Evaluation for potential secondary causes is very important as it plays a crucial role in the management aspect.

## 2. Case Presentation

A 41‐year‐old male presented to the emergency room with a 3‐day history of bilateral leg swelling and skin rash. He also reported fevers with chills and frothy urine. The review of systems was negative. His primary care physician had prescribed furosemide, which did not relieve his symptoms.

His pertinent medical history included human immunodeficiency virus (HIV), which had been diagnosed 16 years prior with the last CD4 count being 1400 cells per μL with undetectable viral load, hypertension, and hyperlipidemia. His medications included atazanavir, lamivudine, zidovudine, rosuvastatin, and valsartan. He was allergic to penicillin with the reaction reported as Stevens–Johnson syndrome. Social history was negative for any recent tobacco, alcohol, illicit, or IV drug use. His sexual history was positive for several same sex and heterosexual contacts. There was no kidney disease reported in family members.

The patient’s vital signs revealed a blood pressure of 145/87 mm·Hg but were otherwise stable. Physical examination was positive for 1 to 2+ pitting edema extending to the knees bilaterally and a maculopapular rash involving hands and soles. The remainder of the examination was unremarkable.

Blood investigations revealed a normal complete blood count and normal renal function panel with a creatinine of 0.8 mg/dL, and his serum albumin level was low at 1.6 g/dL. His rapid plasma reagin (RPR) was positive at 1:128 dilution with confirmatory microhemagglutination assay for Treponema pallidum antibodies (MHA‐TP). Urinalysis showed protein greater than 600 mg/dL and 18 red blood cells per high‐power field with spot urinary protein‐to‐creatinine ratio of 5.7 g/g. Hepatitis B and C serologies were negative. His other immunological workup was unremarkable including normal complement levels, negative anti‐phospholipase A2 receptor (PLA2R) antibody, antinuclear antibody, and anti‐double‐stranded DNA. Radiologic studies revealed normal chest imaging and ultrasound of the kidneys.

**FIGURE 1:**
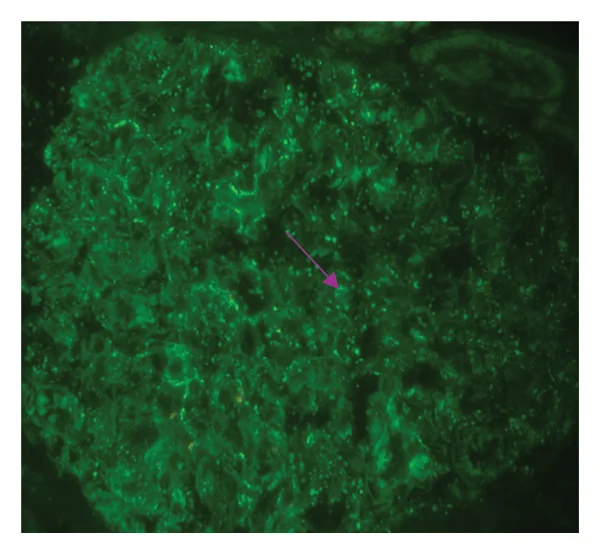
Immunofluorescence with IgG showing positive diffuse granular staining (purple arrow) along the capillary loops.

**FIGURE 2:**
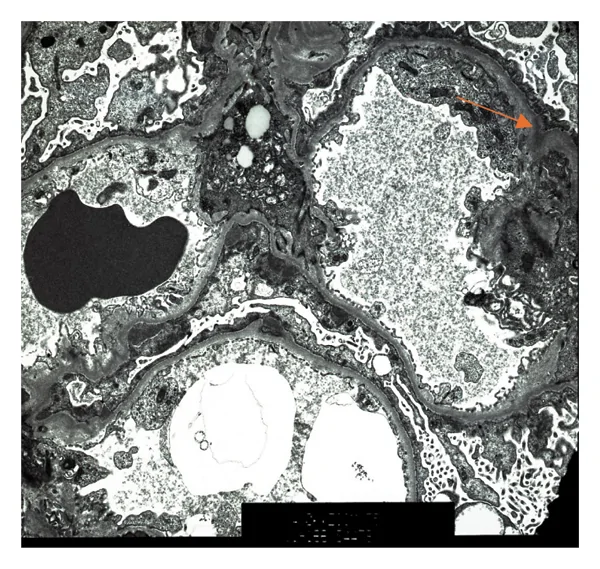
Electron microscopy showed scarce subepithelial electron dense deposits (orange arrow).

He subsequently underwent a percutaneous renal biopsy. Light microscopy showed hyper cellular thick‐walled arterioles with unremarkable glomeruli and no tubulointerstitial changes. Immunofluorescence was positive for abundant granular IgG (as shown in Figure 1) and C3 along the capillary loops. Immunofluorescence for C1q and anti‐PLA2R antibody was negative. Electron microscopy showed scarce subepithelial electron dense deposits as shown in Figure 2.

The patient was started on doxycycline given his penicillin allergy. In addition, the dose of valsartan was increased from 160 mg daily to 160 mg BID for better blood pressure management and control of proteinuria. After a few weeks of treatment, his symptoms and proteinuria resolved completely.

## 3. Discussion

MN is one of the most common causes of adult nephrotic syndrome. The vast majority of cases of MN are idiopathic, while secondary forms of MN are associated with infectious diseases (most commonly hepatitis B and C), autoimmune diseases (most commonly systemic lupus erythematosus), malignancies, and drugs such as NSAIDs. Nephrotic range proteinuria is the most common clinical presentation of MN. Nonnephrotic range proteinuria, microscopic hematuria, hypertension, and renal failure are among the other clinical manifestations. Diagnostic workup of MN should include a thorough evaluation for possible offending medications, infectious causes, autoimmune diseases, and age‐appropriate cancer screening as management strategies depend on causation [1].

Syphilis is now a reemerging disease of public health concern especially over the last decade, with approximately 8 million new syphilis infections in adults aged 15–49 years in 2020, according to recent WHO reports [2]. This is a significant increase compared to the early 2000s, and this trend was noted in both low‐ and high‐resource environments.

Syphilis has varied clinical manifestations [3] and has long been known to be associated with renal disease [4]; however, given the rarity of kidney diseases associated with syphilis infection, most of the data available are limited to case reports. These renal diseases include MN, minimal change disease, focal segmental glomerulosclerosis, and rapidly progressive and crescentic glomerulonephritis (GN) [5–9].

Definitive diagnosis is made by biopsy. The largest cohort of patients with kidney diseases associated with syphilis so far was reported in 2025 and was a retrospective review of the kidney biopsies of 102 patients from 2006 to 2024. The most common disease states included MN, acute tubular injury, and HIV‐associated diseases (collapsing glomerulopathy and immune complex GN) in 29, 24, and 21 patients, respectively, and other less common disease states reported were acute interstitial nephritis, infection‐associated GN, and IgA nephropathy. Of the patients with MN (28.4%), the majority were neuron‐derived neurotropic factor (NDNF) positive and frequently demonstrated staining for multiple immune reactants, particularly C1q. Coinfections including HIV, hepatitis B virus, and hepatitis C virus were common [10]. Our patient did demonstrate staining for IgG and C3 and was coinfected with HIV.

HIV has been associated with a wide spectrum of kidney diseases including HIV‐associated nephropathy (HIVAN) which is characterized by proteinuria and findings of collapsing glomerulosclerosis on renal biopsy, HIV‐associated immune complex kidney disease (HIVICK) in which biopsy reveals immune complex deposits in the kidney, acute kidney injury (AKI), and chronic kidney disease. Patients with HIV are also often coinfected with hepatitis B and/or C which predispose them to nephropathies related to these viruses including MN, membranoproliferative GN, and cryoglobulinemia. In addition, some of the medications used to treat HIV have been associated with varying degrees of nephrotoxicity. Protease inhibitors like atazanavir and indinavir have been associated with crystalluria, urolithiasis, and acute and chronic interstitial nephritis. Nucleoside and nucleotide reverse transcriptase inhibitors including tenofovir are known to cause proximal tubule dysfunction. There have been reports of rhabdomyolysis and AKI due to integrase inhibitors like raltegravir [11]. It is therefore important to consider this wide spectrum of possibilities in HIV patients who present with kidney‐related complaints.

The primary goal of management of syphilis associated MN is treatment of the infection, which in most cases has favorable prognosis on renal outcomes. Other aims of management include treatment of edema with diuretics and optimizing BP and proteinuria reduction with the use of renin–angiotensin system blockers. Literature review of follow‐up renal biopsies after treatment showed resolution of pathological findings in patients who had clinical recovery [12–15].

In our case, the patient was diagnosed with syphilis and its association with nephropathy facilitated the etiological diagnosis of MN. This guided us in the specific treatment of the condition with successful results. Despite its rare occurrence, syphilis should be considered in the evaluation of nephrotic syndrome especially in high‐risk patient populations.

## 4. Conclusion

This case highlights the importance of a detailed assessment in clinical practice for correct diagnosis and appropriate treatment. Syphilis is a rare cause of MN but should be screened for in the appropriate clinical setting.

## Funding

No funding was received for this manuscript.

## Consent

Written informed consent was obtained from the patient for the publication of this case report.

## Conflicts of Interest

The authors declare no conflicts of interest.

## Data Availability

Data sharing is not applicable to this article as no datasets were generated or analyzed during the current study.
